# Vitamin D: Daily vs. Monthly Use in Children and Elderly—What Is Going On?

**DOI:** 10.3390/nu9070652

**Published:** 2017-06-24

**Authors:** Luca Dalle Carbonare, Maria Teresa Valenti, Francesco del Forno, Elena Caneva, Angelo Pietrobelli

**Affiliations:** 1 Clinic of Internal Medicine, section D, Department of Medicine, University of Verona, Verona 37134, Italy; mariateresa.valenti@univr.it (M.T.V.); francesco.delforno@gmail.com (F.d.F.); 2 Pediatric Unit, Verona University Medical School, Verona 37122, Italy; elena.caneva1989@gmail.com (E.C.); angelo.pietrobelli@univr.it (A.P.); 3 Pennington Biomedical Research Center, Baton Rouge, LA 70808, USA

**Keywords:** vitamin D, regimen, children, elderly

## Abstract

Vitamin D deficiency is highly prevalent among children and adults worldwide. Agreement exists that vitamin D deficiency should be corrected. However, the definitions of vitamin deficiency and effective vitamin D replacement therapy are inconsistent in the literature. Not only is the dosing regimen still under debate, but also the time and period of administration (i.e., daily vs. monthly dose). In pediatric as well as elderly subjects, dosing regimens with high vitamin D doses at less frequent intervals were proposed to help increase compliance to treatment: these became widespread in clinical practice, despite mounting evidence that such therapies are not only ineffective but potentially harmful, particularly in elderly subjects. Moreover, in the elderly, high doses of vitamin D seem to increase the risk of functional decline and are associated with a higher risk of falls and fractures. Achieving good adherence to recommended prophylactic regimens is definitely one of the obstacles currently being faced in view of the wide segment of the population liable to the treatment and the very long duration of prophylaxis. The daily intake for extended periods is in fact one of the frequent causes of therapeutic drop-outs, while monthly doses of vitamin D may effectively and safely improve patient compliance to the therapy. The aim of our paper is a quasi-literature review on dosing regimens among children and elderly. These two populations showed a particularly significant beneficial effect on bone metabolism, and there could be different outcomes with different dosing regimens.

## 1. Introduction

The importance of vitamin D in bone metabolism is very well-known. Vitamin D deficiency is associated with increased risk for other non-musculoskeletal chronic diseases [1].

Vitamin D deficiency and insufficiency are highly prevalent among children worldwide [2]. In the adult population, the prevalence of vitamin D deficiency is also high and ranges from 79% to 98% in nursing home patients, increasing the risk of falls and fracture [3,4].

There is still an ongoing discussion to define the optimal 25OH vitamin D (25OHD) serum level for maintenance of bone health, and also what effective vitamin D replacement therapy should involve. In other words, it is not clear which dosing regimens should be used in different populations. However, the evolving consensus is now to recommend a 25OHD target level of 75 nmol/L in fragile elderly subjects who are at elevated risk of falls and fractures, and at least a level of 50 nmol/L for the adult population [3,4].

Thus far, there is no general agreement regarding the dose or the D2/D3 of vitamin D supplementation to reach the target level, even though there are several data showing the different impacts on increasing circulating 25OHD levels in patients on vitamin D supplements, of characteristics such as body mass index (BMI) (accounting up to 30% of variation in circulating 25OHD), type of supplement (vitamin D2 or D3), age, concomitant intake of calcium supplements and baseline 25OHD [5].

The main point is related to the fact that, in clinical practice, maintaining long-term adherence to daily dosages of vitamin D is often difficult, in both pediatric and elderly populations [6]. As a result, more convenient dosing regimens with high vitamin D doses at less frequent intervals were proposed and became widespread practice, despite mounting evidence that such therapies are not only ineffective but potentially harmful, particularly in adults [7].

While it has been shown an association between low 25OH vitamin D levels in childhood and increased occurrence of subclinical atherosclerosis in adulthood [2], definitive data are lacking on the effectiveness of vitamin D supplementation and dose requirements related to the improvement of lower extremity function in elderly. Interestingly, several studies suggested that higher monthly doses of vitamin D increase the risk of functional decline [8,9]. Until now, there are no consistent data suggesting the ideal regimen of supplementation, or comparing daily vs. monthly vitamin D administration.

In light of all the above questions, the aim of our paper is to evaluate dosing regimens among different populations, with particular attention to children and the elderly, to identify the best way to correct vitamin D deficiency, and to define the optimal 25OH vitamin serum level.

## 2. Vitamin D in the Childhood

### 2.1. The First Year of Life

In the first year of life, vitamin D status depends mainly on prophylaxis protocol. Breast milk contains insufficient amounts of vitamin (<80 IU/L) for vitamin D deficiency prevention [10]. Subjects under the age of six months should not be exposed to direct sunlight and in the winter period babies who are exclusively breastfed have lower blood levels of 25(OH)D. To prevent vitamin D deficiency, the American Academy of Pediatrics has recommended a supplement of 400 IU/day from birth for children entirely or partially breastfed [11]. This supplementation is recommended until the child is weaned and drinks at least one liter/day of formula milk fortified with vitamin D. In Italy, Reference Levels of Nutrients and energy intake (LARN) recommend an intake of 400 IU/day between six and twelve months of life [12]. The Italian Society of Paediatrics similarly recommends prophylaxis with 400 IU/day of vitamin D for all infants from the first day of life up to one year of age, regardless of the type of feeding. This dosage is recommended for infants in the absence of risk factors for vitamin D deficiency [13]. Table 1 reports the international recommendations of vitamin D in subjects from birth to one year of age.

#### Daily vs. Monthly Dose

Most clinical studies suggested daily regimens of vitamin D administration. However, daily intake of vitamin D must be supported by sufficient compliance from the family and subjects. Two recent studies instead evaluated the use of prophylaxis with vitamin D in intermittent doses [23,24]. In the first study, 120 children recruited during the vaccination campaign were divided into three groups: 200 IU/day, 400 IU/day, and 50,000 IU every two months. Subjects with 50,000 IU supplementation every two months showed an increase of 25(OH)D higher than the other two groups. At 6 months, 97% of the group of children treated with 50,000 IU every two months reached vitamin D levels ≥ 30 ng/mL (vitamin D sufficient). Only 37% of children in the first group and 77% of children in the second group reached the same level at follow-up. There were no reported cases of hypercalcemia or any other side effects during the study [23]. The second study evaluated 82 breastfed babies born to mothers with vitamin D deficiency. Children were divided into two groups. The first group (children with vitamin D < 20 ng/mL) received 30,000 IU of vitamin D in a single dose in the first week of life, followed by a second dose of 30,000 IU after a month if values of vitamin D were < 20 ng/mL. If at control the values of 25(OH)D levels were > 20 ng/mL, the prophylaxis continued with daily administration of 400 IU/day. The second group was supplemented with 400 IU/day from the second week of life. At follow-up performed after two months, 100% of children supplemented with 30,000 IU in a single dose showed levels of 25(OH)D > 20 ng/mL, with 93.3% having levels > 30 ng/mL. In the second group, 65% had at follow-up levels of 25(OH)D > 20 ng/mL and 27.9% levels > 30 ng/mL. The sufficient level of vitamin D (>30 ng/mL) was achieved in all cases only in children treated with the first prophylactic scheme, while only 50% of children treated with 400 IU/day reached this blood level of vitamin D. Both studied groups reported no side effects due to the administration of vitamin D [24]. Single dose or monthly administered vitamin D would offer greater adherence to prophylaxis by families and children and better efficacy in maintaining or increasing blood levels of vitamin D, in the absence of side effects or toxicity [13].

### 2.2. Children Aged 1–18 Years

Many international studies show that deficit and insufficiency of vitamin D, defined as blood levels of 25(OH)D < 20 ng/mL or between 20 ng/mL and 29 ng/mL, are very common in industrialized countries and in developing countries. A multicenter study conducted in Europe on 1006 children between 12.5 years and 17.5 years found 42% of subjects with deficit and 39% with vitamin D insufficiency [25]. Data collected from Italian children are similar to those from Europeans and Americans, in particular the highest percentages of hypovitaminosis D are found in the neonatal period [26] and adolescence [27,28,29]. Status of vitamin D in infants is influenced by season of birth, ethnicity and maternal prophylaxis during pregnancy, while for adolescents and children important factors include the season in which they get the dosage, sun exposure, ethnicity, and BMI [13]. Important factors in children over one year of age are therefore sun exposure and the presence of certain risk factors for vitamin D deficiency such as non-Caucasian ethnicity, renal or liver failure, intestinal malabsorption, chronic treatment with anticonvulsants, corticosteroids, ketoconazole, antiviral and obesity. The level of recommended daily intake of vitamin D according to the American Academy of Pediatric and Italian LARN for Children between 1 and 18 years is 600 IU/day [30]. The Endocrine Society recommends for children at risk of deficiency an intake of 600 IU/day–1000 IU/day. It is also recommended that obese people receiving anticonvulsants, antiretrovirals, ketoconazole or corticosteroidsare recommended to receive an intake 2–3 times the daily requirements of vitamin D by age [17]. The Society for Adolescent Health and Medicine recommends supplementation with 600 IU/day in adolescents with no risk factors and of at least 1000 IU/day in adolescents at risk for vitamin D deficiency or insufficiency, in addition to vitamin D assumed with diet or produced during sun exposure [31]. These recommendations are important in order to ensure a correct acquisition of bone mass which is known to have its peak in adolescence, a process in which vitamin D is closely involved. Different international health organizations recommend doses of vitamin D on a daily basis, with one or two additional administrations during the winter, with doses of 80,000 to 200,000 IU. The French Society of Paediatrics suggests that children from 18 months to 5 years, with no risk factors for vitamin D deficiency should receive this dosage plus two supplementations of 80,000 IU–100,000 during the winter. The same approach is proposed for adolescents: two similar supplementary doses or a single dose of 200,000 IU during winter [19]. Mallet et al. recommend extending the recommendations for adolescents to children between 6 and 10 years old [32]. Table 2 reports the international recommendations of vitamin D in subjects from 1 year to 18 years of age.

#### Daily vs. Monthly Dose

Some studies that evaluate schedules of vitamin D administration other than daily supplementation offer encouraging results. Carnes et al. evaluated the efficacy and safety of high doses given at intervals every six months for a year. The children were divided into three groups treated with placebo, 150,000 IU every six months or 300,000 IU every six months. The study shows that the best scheme is administration of 300,000 IU/6 months, as this has the highest percentage of patients with normal vitamin D levels. During the study, there were no side effects and compliance remained high [33]. Kuchay et al. showed that intermittent treatment with vitamin D is safe and effective at achieving and maintaining adequate vitamin D levels. The administered dose was 60,000 IU/month for a year. After a year of monthly supplementation, average levels of 25(OH)D had risen from 12.0 ng/mL at the start to 32.6 ng/mL at the end of the study, subjects with insufficiency were reduced from 92.2% at time zero to 2.6% after 12 months, and none of the subjects developed cases of hypercalcemia [34]. In the study of Ghazi et al., 210 children were divided into three groups: one treated with 50,000 IU of vitamin D each month, the second with 50,000 IU every two months and the third with placebo. Treatment with 50,000 IU/month proved effective in increasing vitamin D levels but it does not seem to be enough to correct the deficit, in particular in young girls, who started the treatment with vitamin D levels significantly lower than their male counterparts. (19.25 ± 16.00 vs. 14.00 ± 40.50 nmol/L) [35].

According to the Italian consensus on vitamin D in infants and children [13], vitamin D may be given with intermittent schedule (weekly or monthly for a total dose 18,000 to 30,000 IU/month) from the 5th or 6th year of life to adolescence in those subjects less compliant to daily treatment. Although vitamin D deficiency is detrimental to bone health, the consequent idea that higher doses are protective and confer a reduced risk of disease has been challenged by recent data in adults that indicate that doses of high vitamin D raise the incidence of falls and fractures [36,37]. These events were linked to the mode of administration (i.e., a single large bolus compared to smaller intermittent doses) [38]. Beyond skeletal health, similar curvilinear or U-shaped response has been described for other vitamin D outcomes, including all-cause mortality, cardiovascular disease and selected cancers. For these reasons the Institute of Medicine suggests caution against maintaining serum 25OHD concentrations above 50 ng/mL (125 nmol/L) [39], whereas levels up to 100 ng/mL (250 nmol/L) are cited as safe for both children and adults [17]. The specific vitamin D intake that results in excess or intoxication and the severity of the corresponding hypercalcemia have not been clearly established in pediatric age. Reports on vitamin D intoxication in infants and young children typically describe cases of receiving extremely large doses, in the range of 2.4 million to 4.5 million IU, or approximately 40,000 IU/kg to 560,000 IU/kg. This intake results in serum 25OHD levels in the range of 250 ng/mL–670 ng/mL leading to severe hypercalcemia [40,41]. Serum calcium concentrations in the range of 14–18 mg/dL (normal value: >3.5 mmol/L–4.5 mmol/L) were reported, with occasional values as high as 20 mg/dL. Among these cases, there is significant variability in the amount of vitamin D administered and the resulting serum 25OHD concentrations. Furthermore, even with comparable serum 25OHD levels, the severity of hypercalcemia and symptomatology is unpredictable. Vogiatzi et al. suggest some recommendations for the prevention of vitamin D excess and intoxication in pediatrics [42]. They discourage empirical therapy of vitamin D deficiency with high vitamin D doses (such as single high-dose oral Vitamin D (stoss) therapy) without previous documentation of 25OHD concentrations and monitoring 25OHD and serum calcium levels. Health care providers should also consider monitoring vitamin D levels in infants and children receiving treatment doses at the upper ranges currently recommended. Although there is insufficient evidence to guide the frequency of such testing, Vogiatzi et al suggest 25(OH)D measurements no more than every six months [42]. According to these data, prophylaxis with vitamin D is critical to prevent hypovitaminosis D and the effects on bone metabolism and the whole body. Attention should always be paid to the cumulative dose administered and the risk of toxicity.

## 3. Vitamin D in the Elderly

### 3.1. Elderly Population

Patients over 70 years old represent a frail population in which vitamin D deficiency is frequent and often associated with comorbidities, therefore recommendations for prevention and supplementation of 25OHD are needed to maintain optimal vitamin D levels.

In of our study, we collected the main articles and guidelines published from 2011 to 2016 evaluating efficacy and safety of different vitamin D dosing regimens of supplementations in the elderly population. There is no common agreement worldwide to define a standard dose of cholecalciferol to prevent hypovitaminosis D. Current guidelines from the Institute of Medicine recommends a daily intake of 800 UI, whereas the Endocrine Society Clinical Practice Guideline endorses an intake of 1500 UI–2000 UI daily in people aged 70 years and older [7,43].

Osteoporosis Australia recommends an intake of at least 800 IU in people over 70 years, and even higher doses in specific conditions, such as sun avoiders [44].

In the United Kingdom, instead, people aged 65 years and over as well people with limited sunlight exposure are advised to take daily vitamin D supplements of only 400 IU [45].

A “tailored” dose is likely to be more effective in maintaining vitamin D levels over the therapeutic threshold, because of the high individual variability.

In Italy, the recommendations from the Italian Society for Osteoporosis, Mineral Metabolism and Skeletal Diseases (SIOMMMS) to correct vitamin D deficiency are based on 25OHD baseline levels and require a loading dose to be administered within few weeks plus a maintaining daily supplement dose, similar to the dose regimen suggested by the UK National Osteoporosis Society Practical Guides [45,46].

We did not find relevant results regarding the compliance to the therapy (no studies evaluated the compliance as the primary endopoint), however poor adherence to daily dosing of medications and supplements is reported. In the study by Binkley et al., from 2011, which followed 64 adults over one year, the compliance to daily supplementation was slightly worse compared to monthly supplementation (91–95% vs. 100%) [1].

Another study, by Papaioannou et al., in 2011, showed the adherence to daily vitamin D supplementation of 83% (patients who consumed at least 80% of tablets) [47]. Compliance in real life is expected to be even lower than what is described in literature; a deferred administration of supplements is supposed to be useful to obtain a higher adherence to the therapy and better 25-OHD blood levels.

In the last few years, several trials comparing different daily or monthly dosing regimens of vitamin D currently adopted worldwide resulted in 20–25% of patients having suboptimal vitamin D status (at least 30 ng/mL), even when 50,000 IU were administered monthly (1600 IU/day) [1,47,48]. In addition, the relevant impact on circulating vitamin D levels of variables such as BMI, age or type of supplements will probably lead to a personalized supplementation strategy tailored to the patient. Table 3 reports the international recommendations of vitamin D supplements in the adult population.

Generally, these guidelines are based on 25OHD levels, and do not consider the impact of body weight on increasing circulating 25OHD levels, which accounts for more than 30% of individual variation [5]. 

#### 3.1.1. Vitamin D, Weight and BMI

The influence of weight as well as BMI in conditioning the serum levels of vitamin D has already been evaluated.

Zittermann et al. analyzed the potential determinants of vitamin D levels and found that dose per kg body weight per day could explain 34.5% of variation in circulating 25OHD [5]. Additional significant predictors were type of supplement (vitamin D2 or vitamin D3), age, concomitant intake of calcium supplements and baseline 25OHD levels. Table 4 shows Vitamin D daily supplementation according to weight, age and target 25OHD level 75 nmol/L, considering a baseline 25OHD level 25 nmol/L.

Wijnen et al. underlined that the most effective way to correct vitamin D deficiency in elderly subjects is to administer a personalized loading dose based on body weight and baseline 25OHD levels within few weeks to normalize 25OHD status, then to continue with a supporting dose given monthly (or even every two weeks, in view of the half-life of 25OHD of two weeks) based on body weight [4,5]. This approach seems to be efficient and at the same time safe and able to achieve a satisfying adherence to the treatment of the elderly [4,5]. Due to its clinical practical relevance, as already indicated by the algorithm used in the study from Wijnen et al., it is desirable to include the role of BMI in vitamin D supplementation in further guidelines as well as vitamin D baseline levels [5].

#### 3.1.2. Daily vs. Monthly Dose

To improve adherence of patients to treatment, a deferred regimen is proposed by many authors as a valid alternative to daily treatment. However, there is growing evidence that infrequent high-dose vitamin D supplements might be less effective or even harmful. Pekkarinen et al., in 2009, have shown that supplementing vitamin D every four months is less efficient compared to the equivalent dose in daily administration [50]; moreover, a double-blind, placebo-controlled trial of more than 2000 women aged 70 years or older showed a significant association between annual oral administration of high-dose cholecalciferol and an increased risk of falls and fractures [36].

The effect of a loading dose in addition to daily vitamin D regimen in increasing vitamin D levels was evaluated by Papaioannou et al., and the study showed no significant differences in 25OHD values over three months of treatment, between the groups supplemented with or without a loading dose [47]. This result might have been influenced by the difference of supplements used for loading dose (vitamin D2) and daily administration (vitamin D3).

Meekiins et al., among 39 non-lactating women aged 18 years–40 years old, compared the different pharmacokinetics of a 150,000 IU single dose vs. a 5.000 IU daily dose of cholecalciferol over 28 days, and showed no difference in the cholecalciferol area under the curve between the two groups, although the single dose group had a significantly greater mean of 25OHD [51].

Another study was set to evaluate the effect of daily and monthly dosing of D2 or D3 on circulating 25OHD; the frequency of dosing did not significantly impact 25OHD levels, but the compliance to the treatment was higher in the monthly group. Additionally, the results showed that vitamin D3 is significantly more effective than D2 in increasing serum 25OHD over 12 months [1].

The above-mentioned interesting study from Wijnen et al. in subjects with a mean age of 84 years, compared the efficacy of an individualized cholecalciferol loading dose vs. a daily dose regimen of cholecalciferol 800 IU; the loading dose was calculated according to the algorithm (40 × (25OHD target level − 25OHD baseline level) × body weight), and administered in doses of 50,000 IU twice a week until the total dose was reached [4]. The results showed that patients supplemented with a personalized loading dose reached optimal vitamin D levels within five weeks, with a very large difference in percentage of patients compared to the daily dose group. The administration of a loading dose based on body weight and baseline 25OHD values seems to normalize vitamin D levels in a faster and much more efficient way [4]. It also suggested that the supplementation doses recommended by some of the current guidelines (i.e., Australia Osteoporosis, Institute of Medicine, Endocrine Society Clinical Practice Guidelines) are possibly not effective in correcting vitamin D deficiency in the elderly population.

On the other hand, Bischoff-Ferrari et al. pointed out the controversial role of vitamin D supplementation in the prevention of falls and fractures; the study involved 200 women, selected on the basis of a prior fall, with a mean age of 78 years over one year, divided into three groups based on different monthly vitamin D3 treatments: 24,000 IU/monthly (control group), 60,000 IU/monthly or 24,000 IU/monthly plus 300 mcg calcifediol. Although higher doses of supplements were more effective in reaching reach levels of 30 ng/mL, there were no benefits to lower extremity function; moreover, in the two high-dose groups the risk of falls was significantly increased [8]. This study showed a 5.5 times greater risk of falling in patients reaching the highest quartile of 25OHD level (44.7 ng/mL–98.9 ng/mL) compared with those reaching the lowest quartile (21.3 ng/mL–30.3 ng/mL), suggesting a U-shaped curve (rather than a J-shaped curve) correlation of the effect of vitamin D status on prevention of falls [8]. Further, Smith et al. supported the evidence of a U-shaped curve response to vitamin D3 dose and fall rate and a potential difference between high and medium doses of vitamin D [52].

These results might support the conservative (although not completely effective) approach of IOM recommendations of 800 IU/day of vitamin D3, and cautions against higher doses of vitamin D3, even though they are required to obtain an optimal 25OHD target level [7].

While it is clear that 25OHD levels should be maintained up to a target level of 30 ng/mL, further studies are required to identify a possible upper threshold for the safety of 25OHD levels.

The detrimental effects on efficacy and safeness of high and/or deferred doses of vitamin D supplementation showed by the latest trials are not completely clear and are currently under investigation. The negative outcome on bone health is probably also related to the fact that high doses of vitamin D increase FGF-23 concentration, sclerostin and Dickkopf-1 (DKK-1). The sclerostin and DKK-1 increase inhibits Wnt signaling, and might trigger an upregulation of 1,25-dihydroxyvitamin D3 24-hydroxylase, resulting in increased metabolism and blood levels of 1,25-dihydroxyvitamin D3 [7,49,53].

In the studies we analyzed, no significant differences in hypercalcemia (rarely detected) or urinary calcium levels were found between daily or deferred vitamin D supplements administration [1,47,50,51] (Table 5).

## 4. Discussion

Timing and dosing regimen are crucial for vitamin D supplementation. Daily administration is supposed to be the most physiological way to correct vitamin D deficiency, but a less frequent administration is likely to improve patient compliance to the treatment, and help obtain a greater mean vitamin D 25-hydroxylation [1,51].

In Italy in the first year of life, an intake of 400 IU/day from six to twelve months is recommended [12], even though, as reported in Table 1, different international health organizations give specific and sometimes slightly different recommendations due to different sun exposures and study results. In children and adolescents from 1 to 18 years of age, the recommended daily intake of Vitamin D is 600 IU/day, according to the American Academy of Pediatrics [30]. However, once again, looking at Table 2, different international health organizations give different suggestions for the 1–18 age range. These recommendations come from studies where potentially conflicting factors were considered and results were clearly different. 

Among the elderly too, as reported in Table 3, different international health organizations give different recommendations for Vitamin D supplementation, introducing more confusion. To improve adherence of patients to treatment, a deferred regimen is proposed by many authors as a valid alternative to daily treatment, even though infrequent high-dose vitamin D supplement might be less effective or even harmful. In Italy, in elderly subjects, the current recommendations suggest normalizing 25OHD levels within few weeks with a loading dose based on baseline 25OHD concentration, which is a simplification of the effective dose able to normalize vitamin D levels. Interestingly, some authors suggest the use of a specific algorithm to calculate the loading dose, combining vitamin D levels and weight [4,54]. This approach is interesting because it represents an effort to define a more tailored treatment. From another point of view, the loading dose approach has been criticized by other authors as a cause of increased risk of falls and fractures [7]. Moreover, this negative result emerging from the study by Bischoff-Ferrari might be influenced by the selection criteria (patients with a prior fall) and by the lack of a placebo group. From another point of view, this conflicting result should be explained with the effect exerted by high doses of cholecalciferol that induce an acute increase of FGF-23, and consequently of turnover [53]. Based on these observations, the best approach seems to be a loading dose calculated using the abovementioned algorithm, followed by a daily/monthly maintaining dose, chosen on the basis of the characteristics of the patient. 

## 5. Conclusions

In summary, the best approach to correct a vitamin D deficiency is still debated and could be specific for different age groups. To give clear and practical guidelines, it could be time to set up an expert panel to identify the best dosage of vitamin D at different ages. Many other variables could be included in the analysis to obtain the desirable dosage. 

However, after a critical revision and analysis of all evidence, we could suggest, as key factors, considering bone mass analysis in children and the role of weight or BMI in the elderly in order to define “the Vitamin D picture”. It is also fundamental to ensure a correct acquisition of bone mass which is known to reach its peak in adolescence, a process in which vitamin D is closely involved.

Finally, we can suggest the administration of a loading dose based on body weight and baseline 25OHD values to normalize vitamin D levels, while it is urgent to define the optimal regimen of vitamin D supplementation for maintaining normal levels, having a clear picture of daily vs. monthly administration, and low vs. high dosage. 

## Figures and Tables

**Table 1:** Recommendations for vitamin D prophylaxis in the first year of life according to several international health Societies.

Society	Vitamin D Supplementation
Society of Lawson Wilkins Pediatric Endocrinology (Misra 2008 [14])	- 400 IU/day should be initiated from the first day of life in all breastfed babies, and children not breastfed do not take at least one liter/day of milk formula fortified with vitamin D. - Children with dark skin or who live at high latitudes (>40°) may require vitamin D supplementation at higher doses (800 IU/day), especially during the winter months.
ESPGHAN (Braegger 2013 [15])	400 IU/day of vitamin D in all children during the first year of life.
AAP (Wagner 2008 [11], Golden 2014 [16])	Children breastfed or partially breastfed with 400 IU/day from the first day of life. Supplementation until the child is weaned and takes at least 1 liter/day of vitamin D-fortified milk formula.
Endocrine Society (Holick 2011 [17])	Children in the first year of life at risk of vitamin D deficiency should receive supplementation with 400 IU/day to 1000 IU/day.
Health Canada and the Canadian Paediatric Society [18]	0–6 months - 400 IU/day in breastfed children. - Children who are not breastfed require prophylaxis with vitamin D because infant formula contains vitamin D. - Children partially breastfed 400 IU/day, regardless of how much formula milk taken. 6–12 months - 400 IU/day in children who are still exclusively breastfed or who take breast milk.
United Kingdom Department of Health [19]	- All children between 6 months and 5 years: to ensure a 280 IU/day–340 IU/day intake. Infants fed with formula milk do not require prophylaxis if they take at least 500 mL/day of formulation enriched milk with vitamin D. - Breastfed babies may need to receive prophylaxis from the first month of life if the mother did not take supplements during pregnancy.
Paediatric and Adolescent Bone Group UK (Arundel 2012 [18])	It recommends that children fed exclusively by breastfeeding start prophylaxis immediately after birth.
A French company of Paediatrics (Vidailhet 2012 [19])	- Children fed exclusively by breastfeeding: 1000 IU/day–1200 IU/day for the entire lactation. - Children under 18 months of age who take milk fortified with vitamin D3: 600 IU/day–800 IU/day. - Children under 18 months of age receiving unfortified cow’s milk with vitamin D3: 1000 IU/day–1200 IU/day.
A Spanish company of Paediatrics (Martinez Suarez 2012 [20])	For the child in the first year of life: 400 IU/day or the use of formula milk sufficiently enriched with vitamin D are the best strategies to ensure adequate vitamin intake.
Central Europe (Płudowski 2013 [21])	- Prophylactic vitamin D should start from the earliest days of life, regardless of the type of feeding. - 400 IU/day up to 6 months of life. - 400 IU/day–600 IU/day between 6 and 12 months of life, according to the contribution of vitamin D daily with the diet.
Australia and New Zealand (Paxton 2013 [22])	Children at risk of vitamin D deficiency: 400 IU/day at least for the first year of life.

**Table 2:** Indications for vitamin D prophylaxis between 1 year and 18 years of life according to several international health organizations.

Society	Vitamin D Supplementation
The American Academy of Pediatrics (Wagner 2008 [11], Golden 2014 [16])	Wagner 2008: - Teenagers who do not get 400 IU/day of vitamin D through milk or other fortified foods: 400 IU/day. - Children with increased risk of vitamin D, malabsorption, anticonvulsants treatment: 400 IU/day. Golden 2014: Children over one year of age and adolescents: 600 IU/day, obese subjects treated with anticonvulsant drugs, corticosteroids, antifungal or antiretroviral drugs may require 2–4 times the recommended dose of vitamin D.
Endocrine Society (Holick 2011 [17])	600 to 1000 IU/day. Obese subjects treated with anticonvulsant drugs, corticosteroids, antifungals such as ketoconazole and antiretroviral drugs should receive at least 2–3 times the daily requirements of vitamin D for their age.
ESPGHAN (Braegger 2013 [15])	- UL: 2000 IU/day between 1 year and 10 years old, 4000 IU/day between 11 years and 17 years.
Society for Adolescent Health and Medicine (2013) [32]	600 IU/day (400 IU/day–800 IU/day according to the preparations available on the market) in healthy adolescents, and supplementation with minimum 1000 IU/day in adolescents at risk of vitamin D deficiency.
United Kingdom Department of Health [19]	All children between 6 months and 5 years: 280 IU/day–340 IU/day. No supplement in children receiving at least 500 mL/day of formula milk enriched with vitamin D.
French company of Pediatrics (Vidailhet 2012 [19])	- In children 18 months–5 years: 2 doses of 80,000 IU or 100,000 IU in winter (November to February) - 6 years to 18 years: 2 doses of 80,000 IU or 100,000 IU in the winter (November to February) or a single dose of 200,000 IU.
A Spanish company of Pediatrics (Martinez Suarez 2012 [20])	Daily intake: 600 IU/day
Central Europe (Płudowski 2013 [21])	- Supplementation with 600 IU/day to 1000 IU/day (depending on body weight) of vitamin D and recommended between September and April. - Supplementation with 600 IU/day to 1000 IU/day (depending on body weight) of vitamin D and recommended throughout the year if good cutaneous production of vitamin D is not guaranteed during the summer. - In obese children and adolescents (BMI > 90th percentile for age and sex) supplementation with 1200 IU/day–2000 IU/day (depending on the severity of obesity) of vitamin D between September and April is recommended. - In obese children and adolescents (BMI > 90th percentile for age and sex) supplementation with 1200 IU/day–2000 IU/day (depending on the severity of obesity) of vitamin D throughout the year is recommended. - UL: 2000 IU/day between 1 year and 10 years old, 4000 IU/day between 11 years and 18 years.
Australia and New Zealand (Paxton 2013 [22])	In subjects 1 year–18 years old with risk factors for vitamin D deficiency: 400U/day or 150,000 IU early autumn.

**Table 3:** Indications for vitamin D supplements in the adult population according to the international health organizations.

Society	Vitamin D Supplementation
Institute of Medicine (2010 [43])	600 IU/day, 18 years–70 years old 800 IU/day, over 70 years old
Endocrine Society Clinical Practice Guideline (2011 [49])	1500 IU/day–2000 IU/day, over 19 years old
Osteoporosis Australia (2016 [44])	At least 600 IU/day, under 70 years old At least 800 IU/day, over 70 years old Sun avoiders or people at risk of vitamin D deficiency: 1000 IU/day–2000 IU/day
National Osteoporosis Society Practical Guides (2013 [45])	People aged 65 years and over, people who are not exposed to much sun, pregnant and breastfeeding women: 400 IU/day
Italian guidelines for diagnosis, prevention and treatment of osteoporosis (2015 [46])	Baseline vit. D level < 25 nmol/L: cumulative dose 600,000 IU supporting dose 2000 IU/day Baseline vit. D level 25 nmol/L–50 nmol/L: cumulative dose 400,000 IU supporting dose 1000 IU/day Baseline vit. D level 50 nmol/L–75 nmol/L: cumulative dose 100,000 IU supporting dose 800 IU/day

**Table 4:** Body weight impact on determining optimal vitamin D daily dose [5].

Body Weight (kg)	30-Year-Old Person	70-Year-Old Person
50	42 µg (1680 IU)	24 µg (960 IU)
75	63 µg (2520 IU)	36.5 µg (1460 IU)
100	84 µg (3360 IU)	49 µg (1960 IU)

Calculated daily vitamin D3 dose for achieving a target 25OHD of 75 nmol/L in vitamin D deficient individuals, based on the paper by Zittermann et al. [5].

**Table 5:** Synthetize findings from recent trials on vitamin D dosing regimen.

Study	Design	Efficacy (25-OHD Level > 30 ng/mL)	Safety
2011, Binkley et al. [1]	1600 IU daily vs. 50,000 IU monthly. 64 adults over 1 year.	Similar efficacy. 19% of patients did not reach a 25-OHD level > 30 ng/mL.	No hypercalcemia detected. Similar serum calcium levels.
2015, Wijnen et al. [4]	800 IU daily vs. Loading Dose (40 × (25-OHD target − 25-OHD baseline) × weight) + 50,000 or 25,000 IU monthly (LD). 30 adults over 26 weeks.	Daily group less efficient than LD group (30% vs. 83%).	Not applicable.
2016, Bischoff-Ferrari et al. [8]	24,000 IU vs. 60,000 IU vs. 24,000 IU + 300 ug calcifediol monthly. 200 adults with a prior fall over 1 year.	24,000 IU group less efficient than 60,000 IU and 24,000 IU + calcifediol (54.7% vs. 80.8% and 83.3%).	Significant increasing in falls in 60,000 IU and 24,000 IU + calcifediol groups compared to 24,000 IU group (66.9–66.1% vs. 47.9%).
2011, Papaioannou et al. [47]	50,000 IU + 1000 IU daily vs. 100,000 IU + 1000 IU daily vs. placebo + 1000 IU daily. 65 adults over 90 days.	Similar efficacy. 25% of patients did not reach a 25-OHD level > 30 ng/mL.	Similar, all adverse events judged unrelated to the study treatments.
2010, Pekkarinen et al. [50]	800 IU daily vs. 97,333 IU every 4 months (4M). 40 women over 1 year.	Daily group more efficient than 4M (47% vs. 28%).	Similar increase in urinary calcium.
2014, Meekiins et al. [51]	5000 IU daily vs. 150,000 IU once. 39 women over 28 days.	Similar area under curve for 25-OHD level.	No relevant changes in serum calcium or phosphorus.
